# Supplementary material for: Multivariate white matter alterations are associated with epilepsy duration
Source: Eur J Neurosci. 2020 Dec 11;53(8):2788–803. doi: 10.1111/ejn.15055 (PMC8246988; doi:10.1111/ejn.15055)
Supplement: Supplementary file 1 — Supplementary Material [file EJN-53-2788-s001.pdf]

Supplementary online material

Multivariate white matter alterations are associated with epilepsy duration

Thomas W. Owen[1], Jane de Tisi[3], Sjoerd B. Vos[4,5,6], Gavin P. Winston[3,5,7], John S Duncan[3,5], Yujiang Wang[1,2,3], Peter N. Taylor[1,2,3]

[1]CNNP Lab (www.cnnp-lab.com), Interdisciplinary Computing and Complex BioSystems Group, School of Computing, Newcastle University, Newcastle upon Tyne, United Kingdom

[2]Faculty of Medical Sciences, Newcastle University, Newcastle upon Tyne, NE24HH, United Kingdom

[3]NIHR University College London Hospitals Biomedical Research Centre, UCL Institute of Neurology, Queen Square, London, United Kingdom

[4]Centre for Medical Image Computing, University College London, London, United Kingdom

[5]Epilepsy Society MRI Unit, Chalfont St Peter, United Kingdom

[6]Neuroradiological Academic Unit, UCL Queen Square Institute of Neurology, University College London, London, United Kingdom

[7]Department of Medicine, Division of Neurology, Queen's University, Kingston, Canada

	Univariate associations with epilepsy duration	
	Z-score vs epilepsy duration	
**ROI**	**left-TLE patients**	**right-TLE patients**
**ATR (L)**	-0.334 (0.029)*	-0.079 (0.332)
**ATR (R)**	-0.404 (0.010)*	-0.102 (0.286)
**CG (L)**	-0.146 (0.207)	0.266 (0.933)
**CG (R)**	**-0.460 (0.004)****	-0.075 (0.339)
**CH (L)**	-0.294 (0.048)*	-0.173 (0.168)
**CH (R)**	-0.198 (0.134)	-0.263 (0.070)
**F (L)**	**-0.493 (0.002)****	-0.052 (0.387)
**F (R)**	-0.316 (0.037)*	-0.074 (0.341)
**UF (L)**	-0.376 (0.016)*	0.027 (0.560)
**UF (R)**	-0.428 (0.007)**	-0.067 (0.357)

Table S1 Results of one-tailed Spearman correlation tests between univariate z-scores and epilepsy duration.

Reported are the Spearman ρ estimates and corresponding p-values; ρ (p-value). **ATR:** Anterior thalamic radiation, **CG:** Cingulum gyrus, **CH:** Cingulum hippocampus, **F:** Fornix, **UF:** Uncinate Fasciculus. **L** and **R** correspond to the left and right hemisphere respectively. Significance levels; * indicates $p<0.05$, ** indicates $p<0.01$, *** indicates $p<0.001$. **Bold** indicates significance after multiple comparisons correction.

	Univariate associations with post-operative seizure freedom	
	ILAE1 vs ILAE2+	
**ROI**	**left-TLE patients**	**right-TLE patients**
**ATR (L)**	0.239 (0.406)	2.055 (0.024)*
**ATR (R)**	-0.509 (0.693)	1.792 (0.042)*
**CG (L)**	-0.009 (0.503)	2.026 (0.026)*
**CG (R)**	-0.332 (0.629)	0.721 (0.238)
**CH (L)**	-2.106 (0.978)	0.137 (0.446)
**CH (R)**	-0.094 (0.537)	0.111 (0.456)
**F (L)**	-0.781 (0.780)	1.587 (0.061)
**F (R)**	-1.037 (0.846)	1.759 (0.044)*
**UF (L)**	-0.552 (0.708)	**2.785 (0.005)****
**UF (R)**	-0.777 (0.778)	0.821 (0.209)

Table S2 Results of one-tailed, two sample t-tests assessing the associations between univariate z-scores and post-operative seizure freedom. Reported are the t-statistics and corresponding p-values; T (p-value). Positive t-statistics indicate that larger negative z-scores pertain to ILAE2+ patients relative to ILAE1 patients. Conversely, negative t-statistics indicate the inverse relationship, that larger negative z-scores pertain to ILAE1 patients. **ATR:** Anterior thalamic radiation, **CG:** Cingulum gyrus, **CH:** Cingulum hippocampus, **F:** Fornix, **UF:** Uncinate Fasciculus. **L** and **R** correspond to the left and right hemisphere respectively. Significance levels; * indicates $p<0.05$, ** indicates $p<0.01$, *** indicates $p<0.001$. **Bold** represents significance after multiple comparisons correction.

	Multivariate associations with epilepsy duration		
	Z-score vs epilepsy duration		
Hemisphere	all patients combined	left-TLE patients only	right-TLE patients only
Ipsilateral	**0.482 (1e-5)*** **	**0.493 (0.002)** **	**0.412 (0.009)** **
Contralateral	0.195 (0.058)	0.162 (0.183)	0.202 (0.130)

Table S3 Results of one-tailed Spearman correlation tests between Mahalanobis distances and epilepsy duration. Reported are the Spearman ρ estimates and corresponding p-values; ρ (p-value). **Ipsilateral**: Mahalanobis distance calculated using all ipsilateral ROI's. **Contralateral**: Mahalanobis distance calculated using all contralateral ROI's. Significance levels; * indicates p<0.05, ** indicates p<0.01, *** indicates p<0.001. **Bold** indicates significance after multiple comparisons correction.

	Associations with clinical variables		
	ILAE 1 vs ILAE 2+		
Mahalanobis distance	all patients combined	left-TLE patients only	right-TLE patients only
Ipsilateral	-0.024 (0.490)	1.230 (0.886)	-1.474 (0.077)
Contralateral	-1.242 (0.109)	0.456 (0.674)	**-2.810 (0.004)** **

Table S4 Results of one-tailed, two sample t-tests assessing the associations between Mahalanobis distances and clinical variables. Reported are the t-statistics and corresponding p-values; T (p-value). Positive t-statistics indicate that larger Mahalanobis distances pertain to ILAE2+ patients relative to ILAE1 patients. Conversely, negative t-statistics indicate the inverse relationship, that larger Mahalanobis distances belong to ILAE1 patients relative to ILAE2+ patients. **Ipsilateral**: Mahalanobis distance calculated using all ipsilateral ROI's. **Contralateral**: Mahalanobis distance calculated using all contralateral ROI's. Significance levels; * indicates p<0.05, ** indicates p<0.01, *** indicates p<0.001. **Bold** indicates significance after multiple comparisons correction.

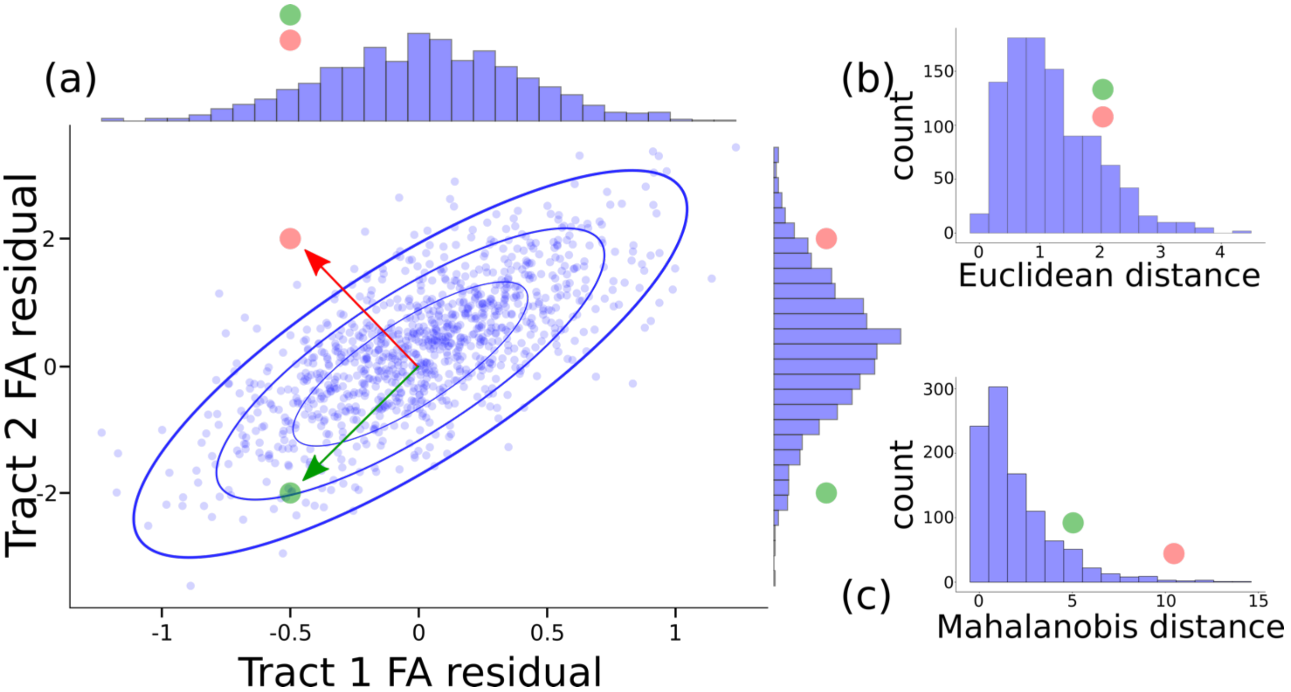

Figure S1 A schematic example demonstrating the importance of accounting for the covariance structure in measures of distance. A population of healthy controls (blue points) covary in their FA values of two tracts **(A)**. If we consider the FA values of two patients (red and green points) we can see that they do not deviate from the controls when considering each tract in isolation (histograms). An extension to the univariate z-score we use the Euclidean distance to discover the shortest path from each point to the center of the control population **(B)**. Here the red and green points deviate from the distribution the same amount and are again within a normal range from the controls. **(C)** Accounting for the covariance structure and penalising points that deviate from it we see a clearer difference between patients and controls. Additionally we see that the red point deviates further from controls than the green point. The approach presented here is visualised in two dimensions for two tracts but can be extended to higher dimensions.

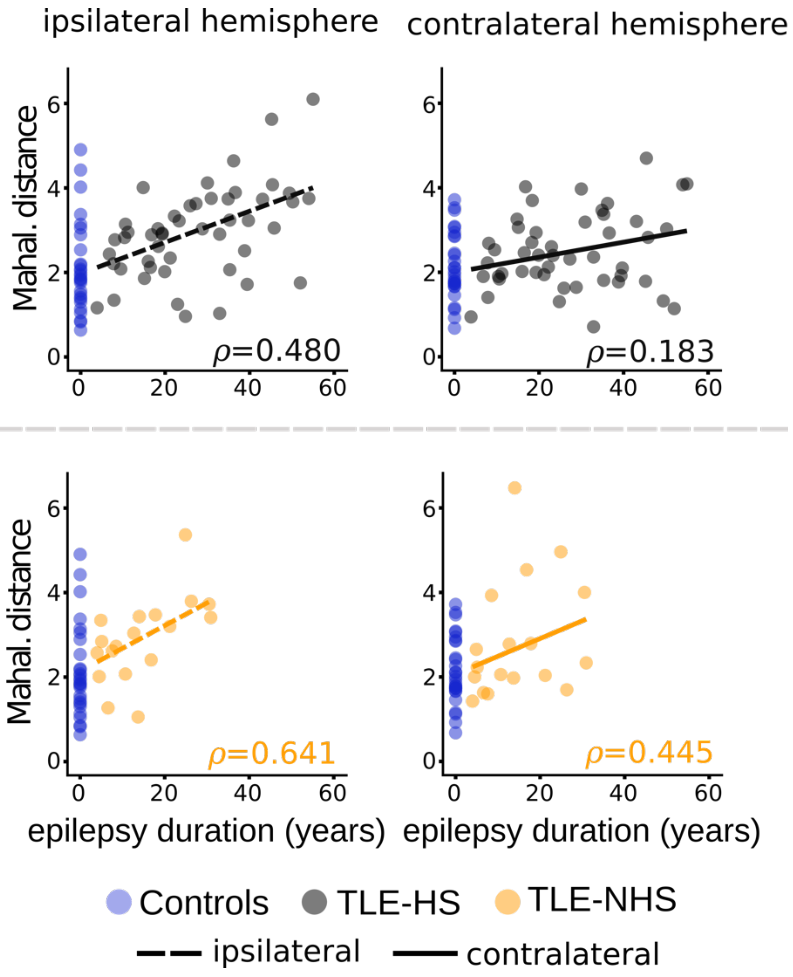

Figure S2 Multivariate associations with epilepsy duration for all ipsilateral and contralateral tract ROI in patients with and without hippocampal sclerosis. Scatter points show the associations between the ipsilateral and contralateral Mahalanobis distances and epilepsy duration for each patient. Here, patients with hippocampal sclerosis (upper panels) and patients without hippocampal sclerosis (lower panels) were analysed separately. Stronger correlations are observed in the ipsilateral hemisphere regardless of pathology. A strong contralateral multivariate association with duration is observed in patients without hippocampal sclerosis only. **Mahal. Dist:** Mahalanobis distance, **TLE-HS:** patients with hippocampal sclerosis, **TLE-NHS:** patients without hippocampal sclerosis

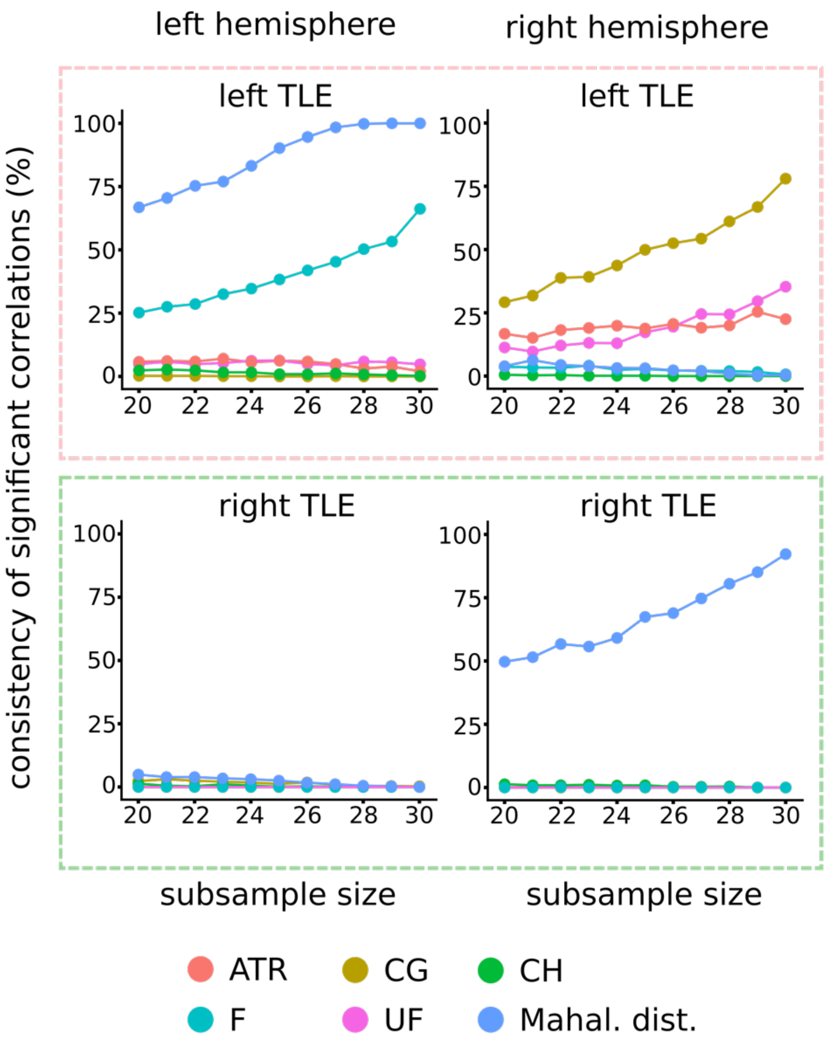

Figure S3 Trajectories of consistency values with varying sample size. Each trajectory corresponds to either a univariate ROI or a Mahalanobis distance. Better consistency values are shown when the subsample size is larger. Consistency scores with high values decay as the subsample size decreases, with a smaller rate of decay exhibited for the Mahalanobis distance. Small consistency values are stable when the subsample size varies. **ATR:** Anterior thalamic radiation, **CG:** Cingulum gyrus, **CH:** Cingulum hippocampus, **F:** Fornix, **UF:** Uncinate Fasciculus, **Mahal. Dist:** Mahalanobis distance.

Simulation study

The aim of the simulation study is to compare the performance of both univariate and multivariate techniques at identifying associations in a noisy dataset, where the underlying structure is known. We randomly generated synthetic data in five independent tracts for 1000 subjects. In each

simulated tract, data for 20% of subjects were correlated with duration providing us with a ground truth association with duration. Each subject is associated with duration in a single simulated tract only.

The results of our simulation study with a ground truth association of moderate strength (between 0.4 and 0.5) can be seen below in figure S3. A univariate approach was unable to identify a strong association with duration in any individual tract when considering the cohort as a whole. This is reflected by the correlation coefficients, of which, the largest in absolute effect size was 0.170. In contrast, the multivariate approach was better able to identify an association with duration in the full cohort, with an effect size of 0.437.

Adjusting the strength of the association with duration in each tract we investigated the performance of the univariate and multivariate approaches at detecting an association in the whole cohort for a range of different values, figure S4. Our results indicate that for a range of ground truth values the Mahalanobis distance outperforms the univariate approach at detecting associations with duration in all instances. When the ground truth value is low the difference between the univariate and multivariate approaches is low but as we increase the ground truth value we see an increased difference between both approaches.

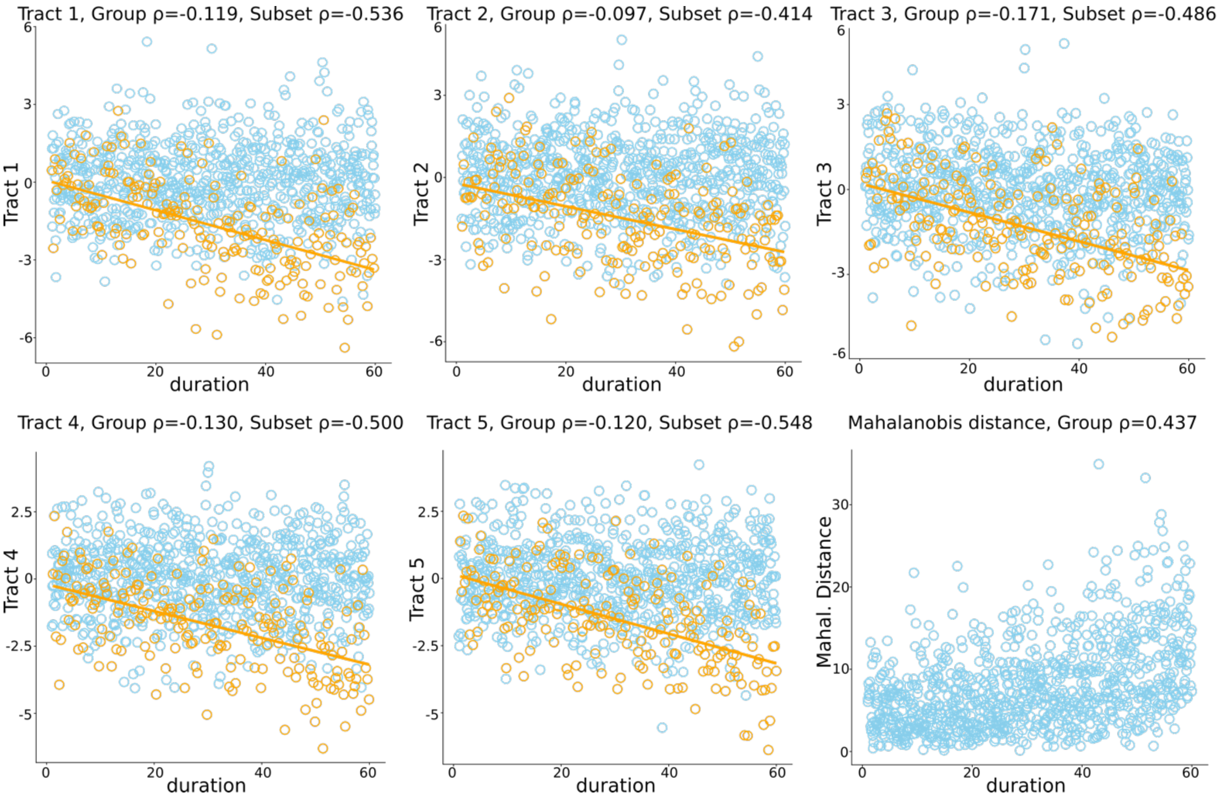

Figure S4 Comparison of univariate and multivariate approaches at detecting associations with duration using simulated data Scatter plots show the univariate and multivariate associations between duration and simulated data for 1000 subjects in five tracts. Blue points correspond to subjects that do not exhibit an association with duration in each tract. Orange points highlight the subset of subjects that do exhibit the imposed association with duration within the tract. Analysis of each tract individually

resulted in small effect sizes when considering the simulated cohort as a whole. Combining all tracts into a single analysis (Mahalanobis distance) shows a stronger association with duration in the whole cohort (rho=0.437) compared to any individual simulated tract.

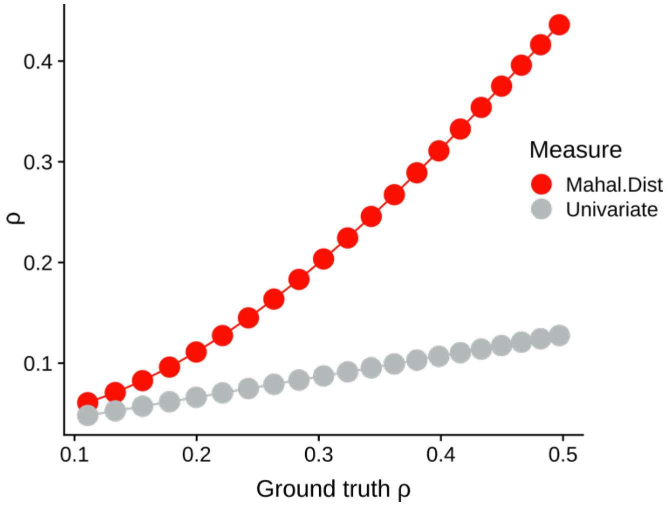

Figure S5 Comparison of univariate and multivariate performance for a range of different ground truth effect sizes. Scatter plot shows the performance of the Mahalanobis distance and univariate approach at detecting associations with duration (y axis) as we alter the magnitude of the ground truth association (x axis). The ground truth is calculated as the average absolute effect size for the subsets of subjects associated with duration in all five tracts. The effect size of the univariate approach is estimated as the average absolute correlation exhibited in all five tracts.
